# Notes and News

**Published:** 1903-12-26

**Authors:** 


### Notes and News.

The Duke of Northumberland has promised to give £1,000 towards building the proposed new infirmary at Alnwick, and has also offered a site.

At the Congress of the Royal Institute of Public Health, to be held at Folkestone in July next year, there will be a section for child study and school hygiene. The local secretary is Dr. Percy Lewis.

The Italian Senate is about to assemble to try one of its members, a well-known surgeon, upon a charge of manslaughter. It is alleged that at the performance of an abdominal operation, a cotton-wool swab was inadvertently left behind in the abdomen, and that this accident resulted in the patient's death.

The sum of £600 bequeathed by the late Dr. Heath to build a residential hall in connection with the Newcastle College of Medicine, is to be devoted to the erection of new physiological and bacteriological laboratories, and a club and smoking-rooms and gymnasium for the students. It is considered that this scheme will be far more useful than the original one.

The fate of the new isolation hospital at Ottawa, which has been opened only a year, should serve as a warning that modern safeguards cannot be disregarded without definitely unsatisfactory results, and that the most careful expert consideration should be given to hospital planning, and that the requirements of the medical staff should above all be taken into consideration. This must have been lacking in Ottawa, for the new institution is already condemned as unfit for its present purpose, as not only is the accommodation for the staff inadequate, all the arrangements have tended to the transmission of one class of disease to the sufferers from another. Consequently, another building altogether is deemed requisite, and the existing hospital is to be retained for scarlet fever and administration only.

Mr Joseph Storer has given a donation of £1,000 to the Bristol General Hospital.

The Chelsea Hospital for Women has received a grant of £105 from the Court of Common Council of the City of London.

The wearisome dispute between the Ballachulish slate quarries and the directors of the company has at last come to an end by the company agreeing to allow Dr. Grant to continue his services. The working men are greatly to be congratulated on the stubborn determination they have shown to prevent an injustice being perpetrated upon their medical attendant.

Several chemists in Paris are being proceeded against for selling morphia without prescriptions. In one instance the prosecutor is a gentleman who claims that as a result of following the advice of a chemist to take hypodermic injections of morphia and cocaine he became addicted to the habit to such an extent as to injure his health. He now claims for £1,200 damages.

At a meeting of the Board of Governors of the Royal Waterloo Hospital for Children and Women, held on the 1st inst., the following resolution was passed:—"That the Board of Governors of the Royal Waterloo Hospital for Children and Women beg to offer their respectful congratulations to H.R.H. the Duchess of Albany on the occasion of the marriage engagement of her daughter Princess Alice to his Serene Highness Prince Alexander of Teck, and to express their best wishes for the happiness of the Princess and the Prince."

The Lord Lieutenant and the Countess of Dudley visited Mercers' Hospital, Dublin, on Thursday, the 17th, and were received by the governors, the lady superintendent, and the medical staff. Having inspected the surgical wards, their Excellencies on arriving at the recreation room examined the hospital records, dating from the year 1734, which includes Handel's music of the "Messiah" first performed in aid of this institution. Subsequently they passed through the medical wards and the children's wards, expressing themselves much pleased with everything they had seen.

The Senate of the University of London have issued an appeal for funds to build and endow an institute of medical sciences under the control of the University. As to the utility of such an institute there can be no question. The present system whereby the preliminary medical sciences are taught in some dozen of schools attached to hospitals is most inefficient. The improvement that would accrue from centralising this teaching in a single institution provided with proper laboratories and apparatus, under the control of sufficiently well-paid and well-qualified teachers, would without doubt not only tend to advance the status of the London medical degree, but also to raise the standard of medical education throughout the country. The question is therefore one which should appeal to all. It is very much to be hoped that some wealthy person will step forward and make a start by placing a substantial sum in the hands of the Senate.

